# 2720. Uninvited Guests in Tiny Tummies: An Assessment of Multidrug Resistant Organism Gut Colonization in Pediatric Transplant Patients

**DOI:** 10.1093/ofid/ofad500.2331

**Published:** 2023-11-27

**Authors:** Simon Parzen-Johnson, Mehreen Arshad, Jacquie Toia, Alima Sajwani, Sameer Patel, Aspen N Kremer, Joseph D Fishbein

**Affiliations:** University of Chicago, Comer Children's Hospital, Chicago, Illinois; Northwestern University/Lurie Children's Hospital of Chicago, Chicago, IL; Ann & Robert H. Lurie Children's Hospital in Chicago, Chicago, IL; Ann & Robert H. Lurie Children's Hospital, Chicago, Illinois; Ann and Robert H. Lurie Children's Hospital, Chicago, Illinois; Ann & Robert H. Lurie Children's Hospital, Chicago, Illinois; Ann and Robert H. Lurie Children's Hospital of Chicago, Hinsdale, Illinois

## Abstract

**Background and Objectives:**

Immunocompromised patients infected with multi-drug resistant (MDR) bacteria are at higher risk of morbidity and mortality. Gut colonization with these strains can increase the risk of invasive infections with the same organism. However, the gut as a reservoir of MDR bacteria is critically understudied in this population. This study therefore aims to determine the prevalence of MDR bacterial gut colonization in pediatric transplant patients and identify associated clinical risk factors.

**Methods:**

A prospective cohort study of pediatric transplant patients was performed at a quaternary medical facility. Patients were enrolled prior to transplant and stool samples were obtained pre-transplant, weekly for one month after transplant, and monthly for the following 5 months. Samples were selectively cultured for ceftriaxone-resistant gram-negative bacilli (CefR-GNB). Immunosuppressant, chemotherapeutic, and antibiotic use was tracked prior to transplant through one year following discharge. Patient demographics, central-line use, admission frequency, and documented infections were also tracked.

**Results:**

Overall, 40 patients have been enrolled with 26 patients having completed the one-year follow-up period. The 26 patients included 11 stem-cell, 5 heart, 7 kidney, and 3 liver transplants. Eleven (42.3%) patients had at least one stool culture with CefR-GNB, and 7 (26.9%) had multiple positive stool cultures. There was no significant association between hospitalizations (P = 0.57), synthetic devices (P = 0.91), median days of gram-negative active antibiotics (P = 0.74), chemotherapeutics (P = 0.64), immunosuppressants (P = 0.43) in the pre-transplant period and risk of colonization with CefR-GNB. One patient developed an invasive infection with a resistant gram-negative organism during the study period.

**Figure:**
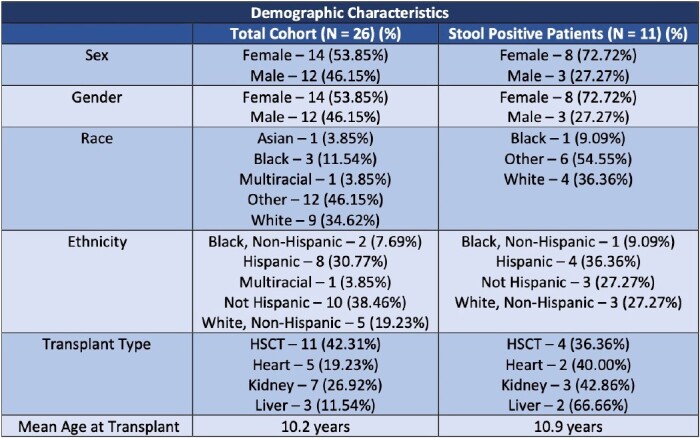

**Figure:**
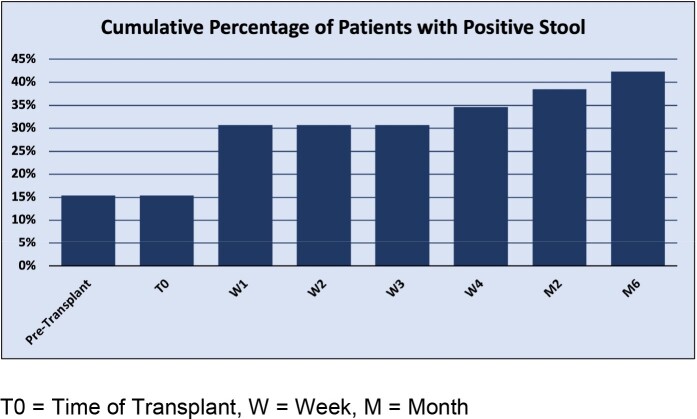

**Conclusion:**

Pediatric transplant patients have high rates of colonization with resistant gram-negative bacilli in their stool. In our small sample size, there was no association seen between prior medication use, admissions, or synthetic devices in place and risk of colonization with resistant organisms. Multi-center studies need to be conducted to assess these risks further.

**Disclosures:**

**All Authors**: No reported disclosures

